# Unilateral Nevoid Telangiectasia in a Female Adolescent: A Case Report

**DOI:** 10.7759/cureus.91560

**Published:** 2025-09-03

**Authors:** Anaisa Afonso, Rita Carvalho, Rui Bajanca, Elsa Lima Teixeira

**Affiliations:** 1 Pediatrics Department, Unidade Local de Saúde da Arrábida - Centro Hospitalar de Setúbal, Setúbal, PRT; 2 Dermatology Department, Unidade Local de Saúde da Arrábida - Centro Hospitalar de Setúbal, Setúbal, PRT

**Keywords:** hormonal skin changes, pediatric dermatology, telangiectasias, unilateral nevoid telangiectasia, vascular skin disorder

## Abstract

Unilateral nevoid telangiectasia (UNT) is a rare, benign vascular disorder characterized by superficial, arborizing telangiectasias distributed unilaterally within a single body segment. The acquired form is the most frequent and is more common in adolescents during puberty and in women of childbearing age, possibly due to estrogen-related mechanisms. Although its true prevalence remains unknown, UNT is believed to be underdiagnosed due to its subtle and often asymptomatic nature. Diagnosis is primarily clinical; however, skin biopsy can assist in confirming the diagnosis and excluding other vascular or dermatologic conditions. We present a case of UNT in a 10-year-old adolescent female, along with a review of the current literature.

## Introduction

Unilateral nevoid telangiectasia (UNT) is a rare, benign vascular skin condition characterized by clusters of superficial, blanchable telangiectasias arranged unilaterally in an arborizing, non-confluent pattern, typically without a central feeder vessel, differentiating it from spider angiomas [1,2]. First described by Zeisler and Blaschko in 1899, its current nomenclature was introduced by Selmanowitz in 1970 [2-4].

UNT can be acquired, which is the most frequent form, or present congenitally. The congenital form, though rare, is more prevalent in males and typically manifests during or shortly after the neonatal period, often persisting into adulthood [5,6]. In contrast, the acquired form is more frequent, predominantly affecting females, and commonly arises during states of hyperestrogenism, such as puberty, pregnancy, or use of oral contraceptives [2,3]. However, it can develop at any age and also occurs in otherwise healthy individuals [4,6].

Clinically, UNT presents as unilateral, red to violaceous, blanchable macules or telangiectatic patches, typically distributed along the C3-C4 dermatomes, but it may also involve thoracic, lumbar, or limb regions [5,6]. Lesions are usually asymptomatic, although cosmetic concerns may prompt dermatologic evaluation [1]. Diagnosis is primarily clinical, based on the distinctive morphology and distribution of the lesions. However, skin biopsy may aid in excluding differential diagnoses, especially in atypical presentations. Histopathologic findings may show dilated, thin-walled capillaries in the papillary and reticular dermis without endothelial proliferation or significant inflammatory infiltrate, helping exclude differential diagnoses such as angioma serpiginosum, hereditary benign telangiectasia, nevus flammeus, hemangiomas, and telangiectasias secondary to systemic disease [1,3-6].

## Case presentation

**Figure 1:**
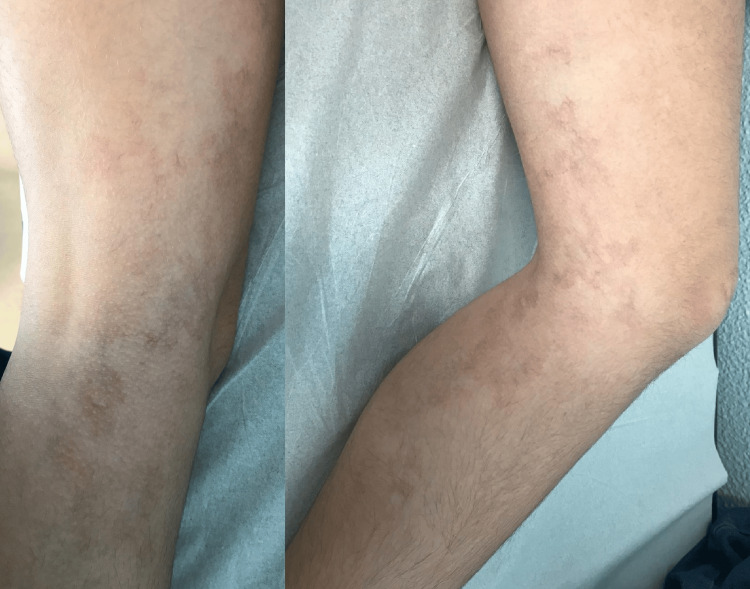
Segmental distribution of unilateral nevoid telangiectasia on the left thigh of a 10-year-old female adolescent

A 10-year-old adolescent with a personal history of atopy and no regular medication was referred to a pediatric appointment for the evaluation of a cutaneous lesion on the left thigh, persisting for several weeks. The lesion was asymptomatic, with no associated pain or pruritus, and gradually became more prominent, with a telangiectatic appearance. On examination, it measured 21 cm at its greatest dimension and exhibited multiple pinkish-brown spots, with irregular borders and a segmental distribution (Figure 1).

**Figure 2:**
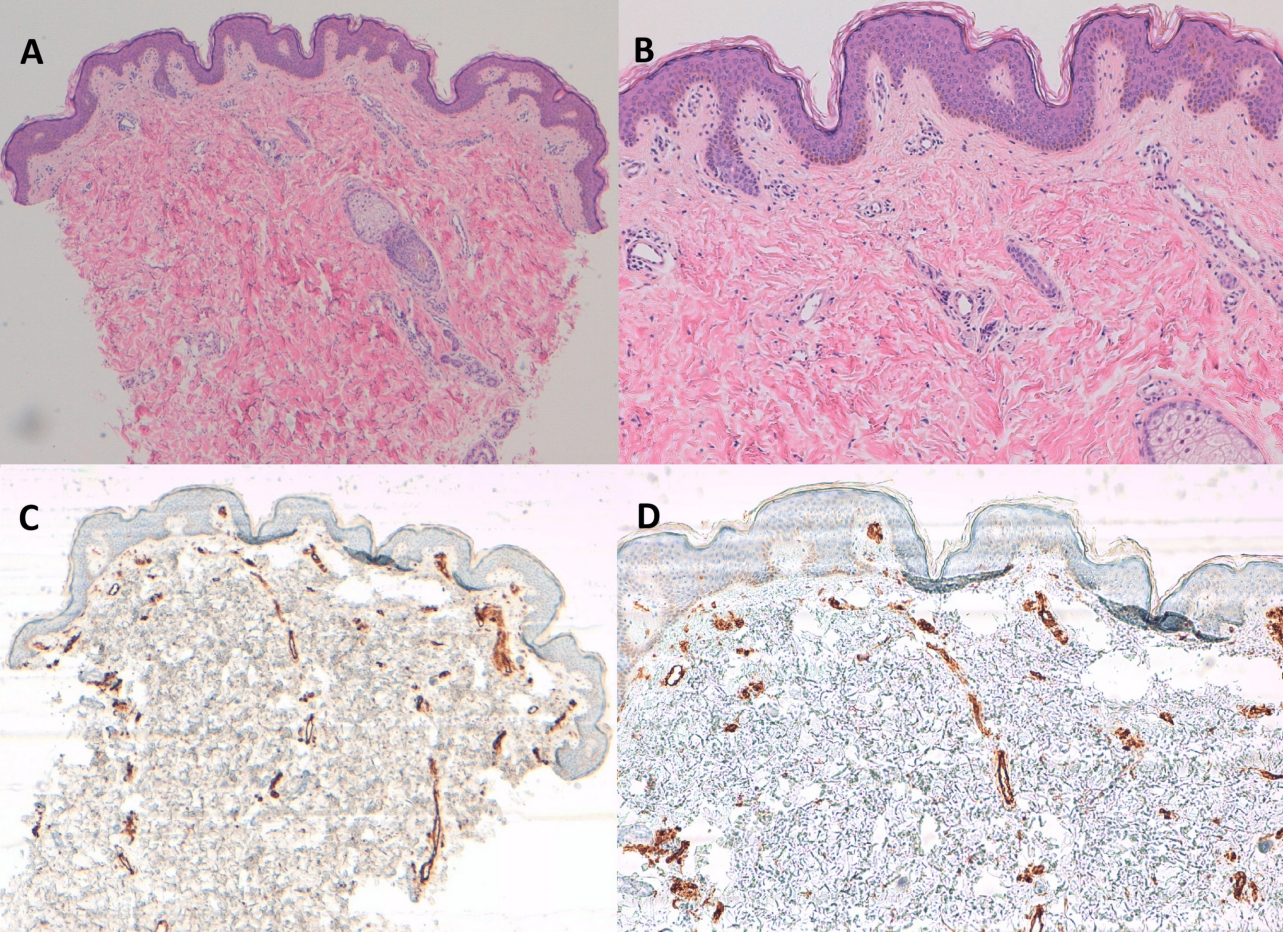
Histopathology of unilateral nevoid telangiectasia in a 10-year-old female adolescent. (A, B) Hematoxylin and eosin (H&E) staining with panoramic and close-up views of dilated thin-walled capillaries in the papillary dermis. (C, D) CD31 immunostaining showing multiple dilated superficial dermal vessels.

The lesion blanched to digital pressure. She was evaluated by dermatology and given the persistence of the lesion over a period of observation, a skin biopsy was performed. Histological examination demonstrated mild changes, including superficial dermal telangiectasias, without endothelial proliferation or significant inflammation, findings consistent with UNT and excluding differential diagnoses such as pigmented purpuric dermatosis (Figure 2).

Complementary laboratory assessment was conducted, revealing normal sex hormone levels for age, negative hepatitis screening, and no evidence of other systemic disease. The adolescent and her family were counseled on the benign nature of the lesion.

## Discussion

UNT is a rare, benign vascular dermatosis that is often underrecognized due to its subtle and asymptomatic presentation and clinical overlap with other vascular conditions. The characteristic unilateral and segmental telangiectasias, particularly in adolescents, should prompt inclusion of UNT in the differential diagnosis [1,3,5].

The pathogenesis of UNT remains incompletely understood. The acquired form, as observed in our patient, is more common than the congenital type and frequently coincides with periods of hormonal fluctuation, such as puberty, pregnancy, or exogenous estrogen exposure. This association supports a potential estrogen-mediated mechanism, and some studies have reported increased estrogen receptor expression [2-5]. However, most patients, including the present case, show no evidence of systemic hyperestrogenism, and histopathological studies often fail to demonstrate overexpression of estrogen receptors in lesional skin, suggesting that hormonal factors alone may not fully explain the condition [2-5,7]. This discrepancy has led to alternative hypotheses, most notably somatic mosaicism, in which postzygotic mutations during embryogenesis lead to localized vascular abnormalities along Blaschko’s lines [2,5]. More recently, pediatric histopathological studies have proposed abnormal estrogen sensitivity in dermatomal endothelial cells, as well as potential involvement of vascular endothelial growth factor (VEGF), particularly in cases linked to liver disease [4,7]. A multifactorial pathogenesis is therefore likely, involving hormonal, genetic, and angiogenic mechanisms [3,4,7]. Accordingly, a comprehensive clinical and laboratory evaluation, including assessment of sex hormone levels, hepatic screening, and thyroid function, is essential to exclude systemic associations [3,6]. In our case, laboratory investigations revealed no abnormalities. Recognizing UNT and distinguishing it from other vascular or dermatologic conditions, such as angioma serpiginosum, hereditary benign telangiectasia, nevus vascularis mixtus, and secondary telangiectasias, is essential to avoid unnecessary interventions and ensure proper management and follow-up [3,6].

UNT is a benign and often asymptomatic condition, and most cases follow a chronic, persistent course, typically managed conservatively. However, due to its aesthetic and psychosocial impact, particularly in adolescents and young women, treatment may be considered and laser therapy remains the gold standard [1,5,7]. Pulsed-dye laser (PDL) therapy is the most effective option for superficial vessels, with consistent success reported in both pediatric and adult patients, reducing or clearing lesions and improving cosmetic outcomes and quality of life [4,5,7]. Nd:YAG (1,064-nm) long pulse laser may also be considered for deeper or larger-caliber vessels [4]. Other options include electrocoagulation, cryotherapy, radiofrequency, and CO₂ laser. In the present case, once the diagnosis was explained and its benign nature clarified, the patient and her family opted for clinical monitoring rather than immediate treatment. Recurrence has been reported, highlighting the importance of long-term follow-up for patients undergoing treatment [4].

## Conclusions

UNT is a rare, benign vascular disorder with a multifactorial pathogenesis involving hormonal, genetic, and angiogenic factors. While primarily a cosmetic concern, timely recognition is essential for accurate diagnosis, exclusion of underlying systemic disease, and discussion of effective treatment options, particularly PDL therapy. UNT should be considered in the differential diagnosis of unilateral vascular lesions, especially in adolescents and women during periods of hormonal change, to optimize patient management and outcomes.
